# Plasticization of Cottonseed Protein/Polyvinyl Alcohol Blend Films

**DOI:** 10.3390/polym11122096

**Published:** 2019-12-14

**Authors:** Wenjie Chen, Jiao Ding, Xuming Yan, Wei Yan, Ming He, Guoqiang Yin

**Affiliations:** 1 College of Chemistry and Chemical Engineering, Zhongkai University of Agriculture and Engineering, Guangzhou 510225, China; chenwj0819@163.com (W.C.); yxmshusheng@foxmail.com (X.Y.); o15112164194@163.com (W.Y.); heming1026@163.com (M.H.); 2 Guangzhou Key Laboratory for Efficient Utilization of Agricultural Chemicals, Guangzhou 510225, China

**Keywords:** cottonseed protein, poly (vinyl alcohol), plasticizer, blend films, triethanolamine

## Abstract

The use of waste plant protein obtained from underutilized and non-food-producing plants may be a promising strategy in the development of bioactive packaging. A series of blend films were prepared by casting from cottonseed protein (CP) and poly(vinyl alcohol) (PVA) and modified with different plasticizers. The morphology, structure, and crystallinity of each of the blend films were characterized by scanning electron microscopy, Fourier transform infrared spectroscopy, and X-ray diffraction. CP and PVA were found to be compatible in the appropriate ratios, and the CP/PVA blend films were partially crystalline. We compared the effects that four plasticizers, i.e., glycerol, triethanolamine (TEA), ethylene glycol, and polyethylene glycol, had on the performance of the CP/PVA blend films. Plasticizers altered the degree of interaction between CP and PVA, which changed the secondary structure of the CP but had little effect on the crystallinity of CP/PVA blend films. Among the different plasticizers tested, TEA was the most efficient at improving the elongation at the break, tensile strength, and oxygen barrier properties of the CP/PVA blend films. Such CP/PVA blend films plasticized using TEA can, therefore, be considered emerging and promising plastics for potential applications in food packaging and flower planting applications.

## 1. Introduction

Synthetic polymer plastics have been widely used in industry and agriculture, thereby leading to environmental pollution and resource shortages. The preparation of biodegradable plastics from renewable biomass resources has, therefore, become a research hotspot, and is expected to aid in the replacement of petroleum-based plastics. During the past decade, low-cost waste biomass, such as feathers, soybeans, corn, and cottonseed meal, have emerged as promising raw materials for biodegradable plastics [1,2,3,4]. Among them, cottonseed meal is considered an appropriate choice.

Cottonseed is an important plant protein resource [5] and is often processed into animal feed [6,7] due to its rich amino acid content and high nutritional value [8,9]. However, cottonseed protein (CP) has not been widely used outside of the food-producition field. This is because the heat treatment process during cottonseed oil extraction causes protein denaturation, which affects the physical and chemical properties and processability of CP. At present, the material application of CP is mainly focused on processing it into an adhesive [10,11]. Throughout the past two decades, few of studies on CP-based films have been published, with the exception of several studies on the compression-molding of CP mixtures using a hot press.

Marquie et al. [12] prepared crosslinked CP films using cotton phenol, formaldehyde, and glutaraldehyde as the cross-linking agents to improve the puncture mechanical properties and water resistance of the films. In further studies, Grevelec et al. [13] prepared glycerol-plasticized CP films and tested their viscoelastic behavior and α-protein relaxation associated with glass transition to determine the optimal process conditions and function of glycerol in enhancing film properties. Their results showed that, when mixed with a higher proportion of glycerol as a plasticizer, the α-relaxation temperature decreased, and the CPs became more stable with regard to thermal denaturation. Moreover, Yue et al. [14,15,16] prepared a series of CP bioplastics (CPBs) starting from glandless CP that was subjected to protein denaturation processes, plasticizing, crosslinking, and hot compression. They investigated the synthetic processing conditions, mechanical strength, and water absorption of the CPBs, and it was found that the use of a glycerol plasticizer increased the ductility of the CP plastic. When the plasticizer content was increased from 10 to 20 wt.%, the elongation at break (EAB) increased from 4.8% to 5.3%. The addition of glycerol also accelerated protein inactivation, as shown by the reduction in the CP inactivation temperature from 156.2 to 136.0 °C in the plasticized CP film.

The mechanical properties of pure CP films are poor, and it is difficult to make them conform to the requirements of packaging plastics even with the addition of plasticizers and crosslinkers. The blending of CP with other polymer materials has, therefore, been performed to improve its mechanical properties and conform to the requirements of packaging materials [17]. For example, poly(vinyl alcohol) (PVA) is a widely used, nontoxic, and biodegradable polymer material with good film formation properties, such that new PVA composites and biomasses are often reported [18,19,20,21,22,23,24]. Chen et al. [25] prepared a feather keratin (FK)/PVA blend film via a casting method, where the EAB and oxygen barrier properties of the blend film increased with increasing the PVA content. In addition, He et al. [26] characterized FK/PVA composite nanofibers fabricated using an electrospinning process. Their results showed that PVA and FK experienced molecular chain entanglement after blending, which improved the spinnability of FK and mechanical properties of the FK-based composite nanofibers. To our knowledge, studies have not yet reported the blend modification of PVA and CP.

Plasticizers can increase the flexibility of polymeric materials. Their efficiency is predominantly governed by their molecular weight, polarity, and compatibility with various materials [27]. However, the effects that different plasticizers have on the properties of CP/PVA blend films have yet to be systematically investigated. We wanted to select a suitable plasticizer to enhance the mechanical and oxygen barrier properties of CP/PVA blend membranes, which are of particular importance to yield promising biomass plastics. We, herein, report the preparation of a series of CP/PVA films via a casting method and the subsequent determination of the optimal ratio of CP to PVA based on the morphological, structural, and mechanical properties of the blend films. To evaluate the effects that different plasticizers have on the mechanical properties, water sensitivity, and barrier properties of the blend films, four plasticizers (including small molecular alcohols, polymeric alcohols, and alcohol amines) were used to modify the CP/PVA blend films. Among these plasticizers, triethanolamine (TEA) effectively improves the elongation at break, tensile strength, and oxygen barrier properties of the CP/PVA blend films, and enhances the application prospects of the CP/PVA blend films as biomass plastics in food packaging and flower planting.

## 2. Materials and Methods

### 2.1. Materials

Cottonseed was purchased from Wuhan Yuancheng Co., Ltd. (Wuhan, China). The plasticizers, including glycerol (Gly), triethanolamine (TEA), ethylene glycol (EG), and polyethylene glycol (PEG, molecular weight: 400), were purchased from Shanghai Macklin Biochemical Co., Ltd. (Shanghai, China). PVA (degree of polymerization: 1700; degree of alcoholysis: 99) was obtained from Shanghai Aladdin Biochemical Technology Co., Ltd. (Shanghai, China); sodium hydroxide and hydrochloric acid were purchased from Tianjin Damao Chemical Reagent Co., Ltd. (Tianjin, China). All commercial reagents were of analytical grade and used without further purification. Deionized water was used as a solvent.

### 2.2. Extraction of Cottonseed Protein (CP)

CP was extracted according to the procedure reported in Zhang et al. [28] with slight modifications. The cottonseed was added to a 0.05 mol·L^−1^ solution of sodium hydroxide (1:12, w/w), and protein extraction was performed at 60 °C for 60 min under stirring. The extraction solution was centrifugated at 3000 rpm for 10 min, after which the centrifuged solution was passed through a 200-mesh nylon filter. The filtrate obtained was subjected to dialysis (Oso-T8280 dialysis tubes, 8000–14,000, Union Carbide, Houston, TX, USA) against distilled water at 25 °C over a period of one week. The CP solution was collected after dialysis, and the pH was adjusted to between 4.7 and 4.9 with dilute hydrochloric acid until there was significant protein precipitation. After precipitation, filtration, and separation, the CP was freeze-dried.

### 2.3. Preparation of the CP/PVA Films

CP/PVA blend films were prepared using a casting method. A 6 wt.% CP solution was adjusted to a pH of 10 with a 0.1 mol·L^−1^ sodium hydroxide solution and then heated at 40 °C for 10 min under continuous stirring. CP/PVA film-forming solutions containing various weight ratios of the 6 wt.% PVA aqueous solution were prepared in which the total CP and PVA solid content (1.8 g) did not change. This was followed by slow stirring for 30 min at 40 °C. The film-forming solutions were poured into polypropylene molds and placed horizontally in a constant temperature and humidity environment (25 °C, 50% RH) to dry. We refer to the CP/PVA solutions and blend films as C_x_P_10−x_, where x is the weight percentage/10 of CP relative to the total weight of CP and PVA in the film. For example, C_3_P_7_ refers to a CP:PVA weight ratio of 3:7.

### 2.4. Preparation of the Plasticized Films

The C_3_P_7_ solution was used to investigate the effect that the plasticizers have on CP/PVA film properties. The 6 wt.% CP and PVA solutions were used to prepare a mixed solution with a CP to PVA weight ratio of 3:7. The CP/PVA solution was magnetically stirred with 5%, 10%, or 20% plasticizer for 30 min at 40 °C to obtain a homogeneous solution. The plasticizers used in this study were Gly, TEA, EG, and PEG. The plasticized CP/PVA film-forming solution was then poured into a polypropylene mold and placed horizontally in a constant temperature and humidity environment (25 °C, 50% RH) to dry. We refer to the plasticized films as n-M, where n is the weight percentage of the plasticizer relative to the total weight of the CP and PVA in the film and M is the type of plasticizer. For example, 20%-TEA refers to a TEA content of 20% relative to the total weight of CP and PVA.

### 2.5. Characterization

The surface morphologies of the films were observed using scanning electron microscopy (SEM, EVO18; Carl Zeiss, Jena, Germany) with a secondary electron detector at an accelerating voltage of 15 kV.

The Fourier-transform infrared (FTIR) spectra of the films were recorded using a Spectrum 100 infrared spectrometer (Perkin-Elmer, Fremont, CA, USA) in attenuated total reflectance (ATR) mode. Scans were recorded from 4000 to 650 cm^−1^ and the combination of four scans was used for analysis. The spectra were analyzed using the Origin 2017 software. The FTIR spectra in the region from 1700–1600 cm^−1^ were fitted using deconvolution and fit-Gaussian to divide the overlapping peaks. The fitting operation was performed multiple times. Finally, the relative percentage of CP secondary structures was calculated according to the integrated area of each fitted peak [29,30,31,32].

X-ray diffraction (XRD) patterns of the samples were analyzed using a D8 X-ray diffractometer (Rigaku, Akishima, Japan) at a voltage of 40 kV and current of 30 mA using Cu-Kα radiation. The 2θ scan range was from 10° to 50° at a scan rate of 2°/min.

The mechanical properties of the films, such as the tensile strength (TS) and EAB, were measured on a microcomputer-controlled electronic universal testing machine (CMT6503, Shenzhen MTS Test Machine Company Ltd., Shenzhen, China). A strain rate of 10 mm/min was used throughout the experiment in accordance with the ASTM standard D638 [33]. We performed three replications of each sample and calculated the average and standard deviation values of the measurements.

The moisture sensitivity of each blend film was evaluated using an automatic contact angle meter (Theta, Biolin Scientific Ltd., Espoo, Finland) to determine the contact angle between the water droplets and the films and the time of the sudden infiltration point. For this, the films were cut into samples measuring 40 × 10 mm, and both ends of the film were fixed to glass slides. Droplets of deionized water were extruded from a needle tube onto the surface of the intermediate suspended film. The contact angle between the film sample and water droplets was continuously measured until the water droplets completely penetrated the film.

The water vapor permeability (WVP) of each film was measured using a water vapor transmittance tester (W3/030, Labthink Ltd., Jinan, China) at 38 °C with a gradient of 90%–0% relative humidity across the film.

The oxygen permeability (OP) of each film was measured using an oxygen permeability tester (VAC-VBS, Labthink Ltd., Jinan, China) at 25 °C, 50% RH with a test gas pressure of 1.01 × 10^5^ Pa and upper and lower degassing times of 4 h.

The thicknesses of each film were measured using a digital external micrometer (accurate to 0.001 mm). The measurements were conducted in triplicate, and average values were calculated.

## 3. Results and Discussion

### 3.1. Film Morphology

**Figure 1:**
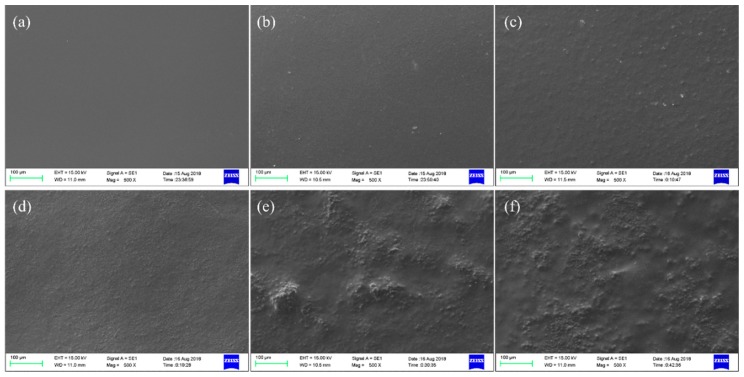
Surface morphologies of the cottonseed protein (CP)/PVA blend films: (**a**) C_1_P_9_, (**b**) C_2_P_8_, (**c**) C_3_P_7_, (**d**) C_4_P_6_, (**e**) C_5_P_5_, and (**f**) C_6_P_4_.

Figure 1 shows the SEM images of the CP/PVA blend films containing different CP-to-PVA mass ratios. The surfaces of the CP/PVA blend films appear smooth with a CP content <40% and no phase separation. However, a large number of small particles agglomerated and precipitated on the surfaces of C_5_P_5_ and C_6_P_4_, which roughened the film’s surface, demonstrating a limited compatibility between CP and PVA. Upon blending the PVA with a small amount of CP, we formed a uniform and stable CP/PVA solution. In this case, the film-forming process was relatively stable. However, upon increasing the proportion of CP, the spaces between the molecules of the film-forming solution increased due to the use of different CP/PVA blend compositions, which, in turn, led the surface molecules to experience different levels of tension during film formation. This affected the flatness of the film surface, forming an uneven and rough surface.

**Figure 2:**
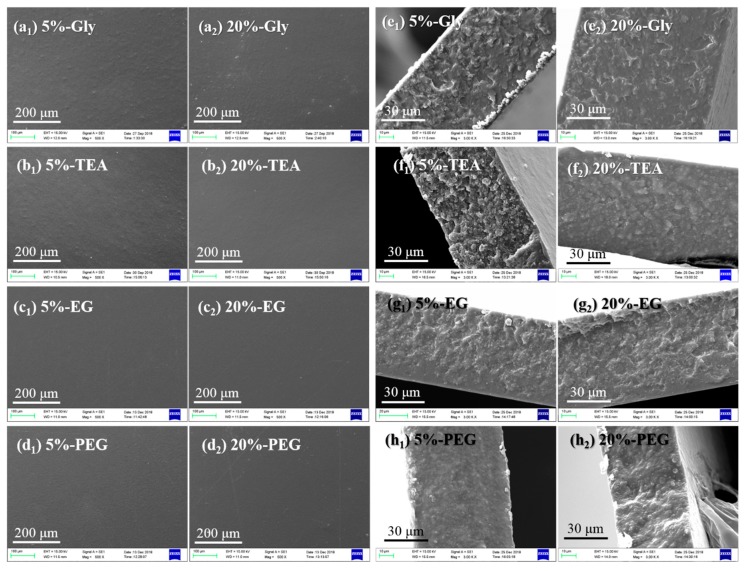
SEM images of the CP/PVA blend films containing different plasticizers and plasticizer contents (5% or 20%): (**a**–**d**) surface morphology and (**e**–**h**) cross-sectional images.

A series of plasticized CP/PVA blend films were then prepared by fixing the CP:PVA mass ratio at 3:7 and adding either a 5% or 20% plasticizer (i.e., the highest and lowest levels). The SEM images of the plasticized CP/PVA blend films are shown in Figure 2, where it is apparent that the surfaces of the plasticized CP/PVA blend films were flat, and the cross-sections were smooth and continuous. This was due to the addition of the plasticizers, which can also interact with the CP and PVA molecules through hydrogen bonding to improve compatibility between the two materials.

### 3.2. FTIR Spectroscopy

**Figure 3:**
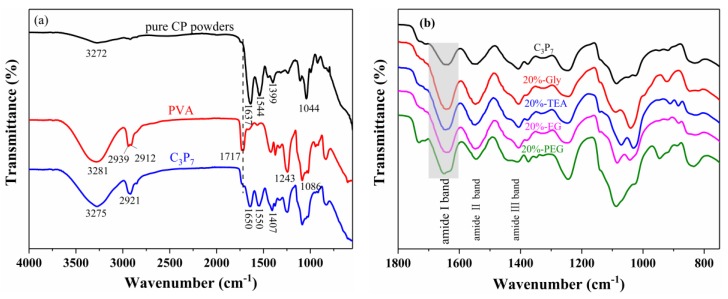
FTIR spectra of (**a**) the raw materials and blended films, and (**b**) the plasticized CP/PVA blend films.

The FTIR spectra of the CP powders, PVA, and CP/PVA blend films (C_3_P_7_) are shown in Figure 3. In the case of the CP powder, a broad and weak band was observed at ≈3272 cm^−1^ corresponding to the O–H and N–H hydrogen-bonding association peaks. This may be due to the fact that the number of amino groups in the CP molecular structure exceeded the number of hydroxyl groups, thereby resulting in a smaller degree of association between N–H and O–H. Furthermore, multiple characteristic absorption peaks of CP were detected in the 1800–1000 cm^−1^ range [33]. For example, the peaks at 1637, 1544, 1399, and 1044 cm^−1^ corresponded to the characteristic absorption peaks of the CP amide I band (i.e., the C=O stretching vibration), the CP amide II band (i.e., the N–H bending and C–N stretching vibrations), the CP amide Ш band (i.e., the C=O bending and C–N stretching vibrations), and the C–O stretching vibration of CP, respectively [15]. Moreover, in the FTIR spectra of PVA, peaks at 3281 (hydroxyl groups), 2939, and 2912 (the asymmetrical and symmetrical stretching of C–H, respectively), 1717 (C=O stretching vibration), 1243 (C–H wagging vibrations), and 1086 cm^−1^ (C–O stretching vibration) were observed [34,35].

In terms of the blend films, a broad band was observed at ca. 3700–3100 cm^−1^—which corresponds to the N–H and O–H hydrogen-bonding association peaks of C_3_P_7_—whose intensities were greater than those of the O–H and N–H association peaks of the CP powder. This may be attributed to the fact that the addition of PVA increased the hydroxyl (–OH) content of the blend films and thereby enhanced the degree of association between N–H and O–H. In addition, the amide characteristic absorption peaks of CP at 1637, 1544, and 1399 cm^−1^ were shifted to 1650, 1550, and 1407 cm^−1^ (corresponding to the amides I, II, and Ш, respectively) in C_3_P_7_, respectively, and the peak intensity was lower than that of the corresponding characteristic peak of CP. This suggests the presence of hydrogen bonding between the CP and PVA molecules. Furthermore, the characteristic peak of the ester carbonyl group of C_3_P_7_ originating from the PVA molecule (1717 cm^−1^) was reduced in intensity, likely due to its partial acylation with the free primary amine group in the CP to form an amide group. This, in turn, likely altered the CP secondary structure in the blend films.

When the small-molecule plasticizer and large-molecule polymer were dissolved, the interaction between the polymer macromolecules (i.e., hydrogen bonding between CP and PVA) can be weakened by establishing a new interaction with the polymer (i.e., hydrogen bonding between the plasticizer and either CP or PVA). This change was beneficial to the macromolecular chain mutual rearrangement (inner rotation) under the action of an external force field (i.e., mechanical force) to the blended film, which improved the toughness and ductility of the polymer films [27]. Figure 3b shows the FTIR spectra of the plasticized CP/PVA blend films at 1800–750 cm^−1^, where the characteristic absorption peaks of amides I, II, and Ш in the plasticized C_3_P_7_ film can be observed at approximately 1650, 1550, and 1407 cm^−1^. The positions and intensities of these peaks differed compared with those of C_3_P_7_, which indicates that no reaction occurred between the plasticizers and CP/PVA mixtures, with the presence of only physical interactions.

**Figure 4:**
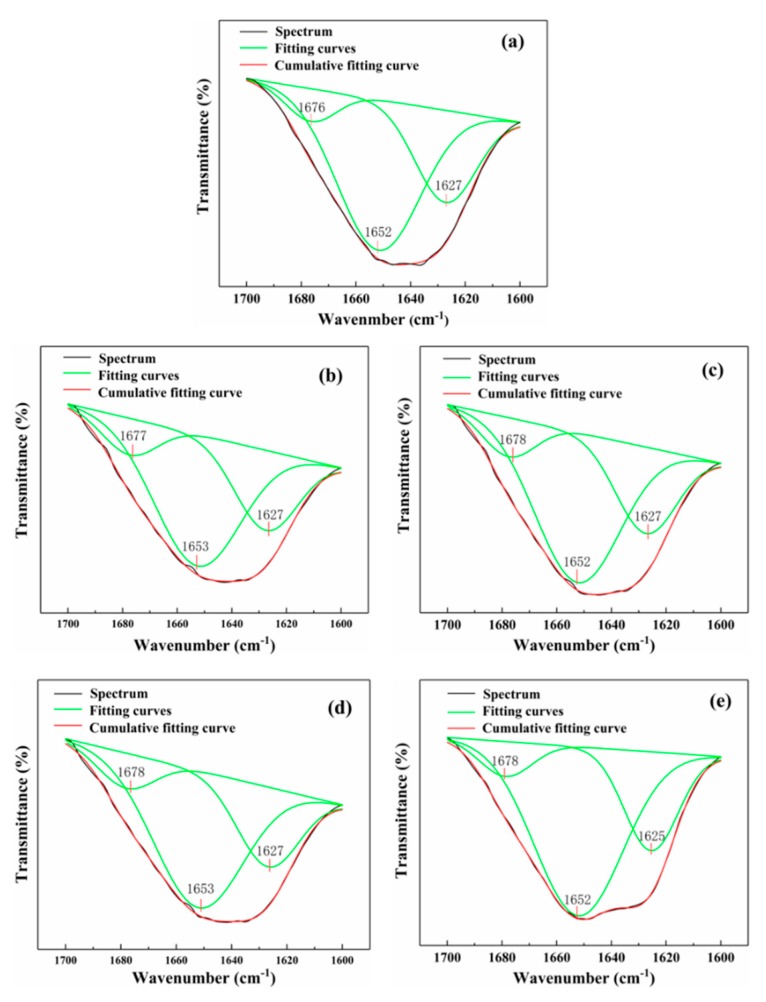
Amide I band spectra of C_3_P_7_ and plasticized CP/PVA blend films: (**a**) C_3_P_7_, (**b**) 20%-Gly, (**c**) 20%-TEA, (**d**) 20%-EG, and (**e**) 20%-PEG.

The amide I band is known to be especially sensitive to any conformational changes in the secondary structure of proteins [33]. Thus, the absorption bands of the amide I band are shown in Figure 4, where the predominant absorption band at ≈1650 cm^−1^ is characteristic of the α-helical structure, and the bands between 1610–1633 cm^−1^ and 1675–1695 cm^−1^ can be attributed to β-sheet and β-turn structures, respectively [26,36,37]. The calculated peak areas of the corresponding structures are summarized in Table 1. The results show that the secondary structure of CP in the blend films consists mainly of α-helix and β-sheet structures, with a small number of β-turn structures also present. The area associated with the β-sheet showed a decreasing trend in the amide I band of the blend film after the addition of plasticizers, whereas there was an increase in the area associated with the β-turn structure. This was attributed to the fact that the plasticizers could gradually be inserted into the CP β-sheet structure to generate hydrogen bond interactions, increasing the free volume of the peptide chain. Plasticizer insertion, therefore, gradually weakens or even breaks the hydrogen bond interactions between the β-sheet layers, resulting in a gradual shift in the β-sheet to a β-turn [38]. This increase in the number of β-turn structures was beneficial in terms of improving the flexibility of the protein-based films. Moreover, there was also an increase in the α-helix content for 20%-TEA, indicating that the addition of TEA can promote the partial conversion of the CP structure to yield a spiral. This rendered the overall structures of the blend films more stable and ductile.

### 3.3. XRD Analysis

**Figure 5:**
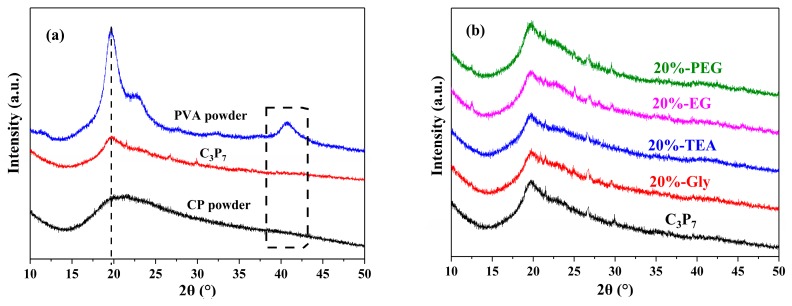
XRD patterns of (**a**) the raw materials and blended films and (**b**) the plasticized CP/PVA blend films.

The XRD patterns of the pure CP powder, pure PVA powder, and CP/PVA blend films between 10° and 50° are shown in Figure 5a. More specifically, the pure PVA powder exhibited two characteristic crystalline peaks at 19.6° and 22.5°, which were attributed to the 101 and 002 crystal planes, respectively [34,39]. Furthermore, a diffraction peak was also observed at 40.7°, indicating that PVA is a semi-crystalline polymer, whose crystallinity is due to strong intermolecular interactions between the PVA polymer networks [25]. In addition, the pure CP powder exhibited a broad diffraction peak at 20.5°. However, for the CP/PVA blend film (C_3_P_7_), two diffraction peaks originated from the PVA component at 22.5°, and 40.7° disappeared, resulting in the broadening of the diffraction peak at 19.6°. These observations confirmed that a strong hydrogen bond formed between the CP and PVA molecules, which destroyed the regularity of the original molecules and affected the crystal structures of both CP and PVA. This is in good agreement with the results of the FTIR analysis.

The XRD patterns of the plasticized CP/PVA blend films are shown in Figure 5b. The diffraction peak of the plasticized CP/PVA blend films appeared at 19.6° and did not shift or broaden compared with the C_3_P_7_ case. Furthermore, no new signals formed between 10° and 50°, which confirmed that the plasticizers did not affect the regularity of the CP/PVA blend films’ crystal structures.

### 3.4. Mechanical Properties

**Figure 6:**
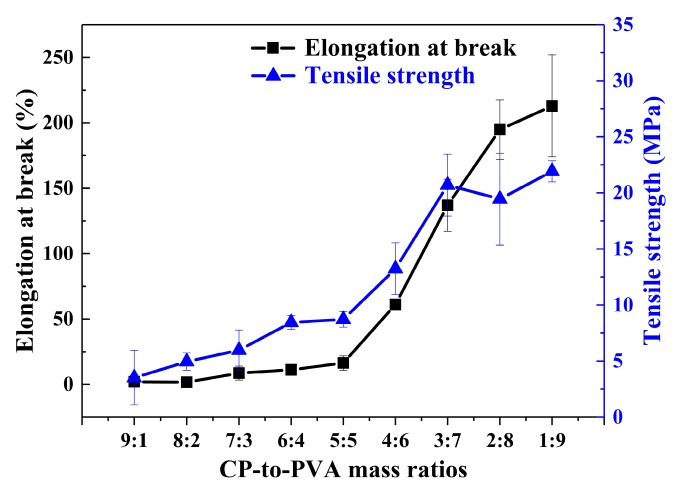
Elongation at break (EAB) and tensile strength (TS) results for the blend films containing different CP:PVA mass ratios.

The main purposes of blending CP with a synthetic polymer are to address issues associated with pure CP, such as poor film formation and brittleness, and to improve the mechanical properties of the films to expand their application prospects. We, therefore, investigated the effects that different CP and PVA mass ratios have on the TS and EAB values of the blend films, whose results are presented in Figure 6. The EAB and TS values of the prepared blend films increased from 2% and 3.5 MPa to 137% and 20.7 MPa, respectively, when the CP:PVA mass ratio was changed from 9:1 (C_9_P_1_) to 3:7 (C_3_P_7_). Although the mechanical properties of pure protein films were generally poor due to their complex elemental compositions, hydrophilic groups, impurities, and sensitivities to heat and moisture, the good compatibility for an appropriate ratio between CP and PVA resulted in their blend films exhibiting improved mechanical properties. Based on this analysis of the microstructural and mechanical properties of the CP/PVA blend films, we determined that the optimal mass ratio for the CP/PVA blend film was 3:7.

**Figure 7:**
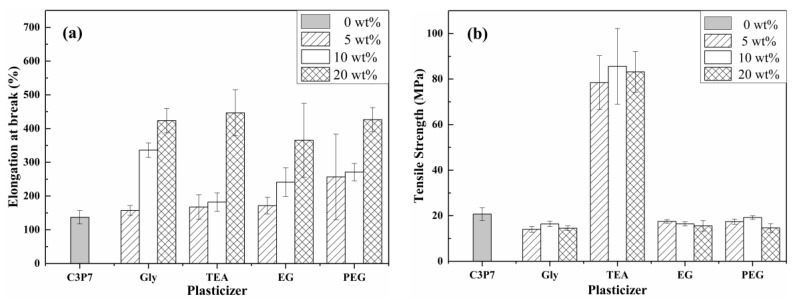
Mechanical properties of the plasticized CP/PVA blend films: (**a**) EAB and (**b**) TS.

The EAB values of the plasticized CP/PVA blend films are shown in Figure 7a, where EAB increased with an increase in the plasticizer content. More specifically, a plasticizer content of 5% promoted the EAB of the CP/PVA blend film by three to five times, whereas a 20% plasticizer addition yielded a seven to nine-fold enhancement. In addition, by comparing the effects that different plasticizers have on the EAB of the CP/PVA blend films, PEG had a better plasticizing effect on the CP/PVA blend films than the other plasticizers (i.e., Gly, TEA, and EG) when the amount of plasticizer added was lower (i.e., 5%). This was attributed to PEG’s larger molecular weight and molecular chain length compared with the other three plasticizers, which allowed it to increase, to a greater extent, the space between macromolecular chains. This, in turn, promoted a greater relative slidability on macromolecular segments when the PEG molecules were inserted between the CP/PVA macromolecular chains than when using other plasticizers. However, the degree to which the type of plasticizer influenced the EAB of the CP/PVA blend films was smaller than the influence of the amount of plasticizer added.

The tensile strengths of the plasticized CP/PVA blend films are shown in Figure 7b. As indicated, only TEA increased the TS of the CP/PVA blend film, while Gly, EG, and PEG had no effect. More specifically, 10%-TEA gave a TS of 85.60 MPa, which was four times that of C_3_P_7_. This increased TS may be due to the presence of a more electronegative tertiary amine group in the TEA molecule, which can form an intermolecular hydrogen bond between the H atoms on the N and O atoms in the CP/PVA macromolecular chain. However, this tertiary amine group can also partially enhance the intensity of hydrogen bonding in the H atom on α-C [40,41,42]. In this case, a new network structure formed among the TEA, CP, and PVA molecules, which inhibited movement between molecules while increasing the tensile strength of the CP/PVA blend film.

### 3.5. Moisture Sensitivity of the Blend Films

**Figure 8:**
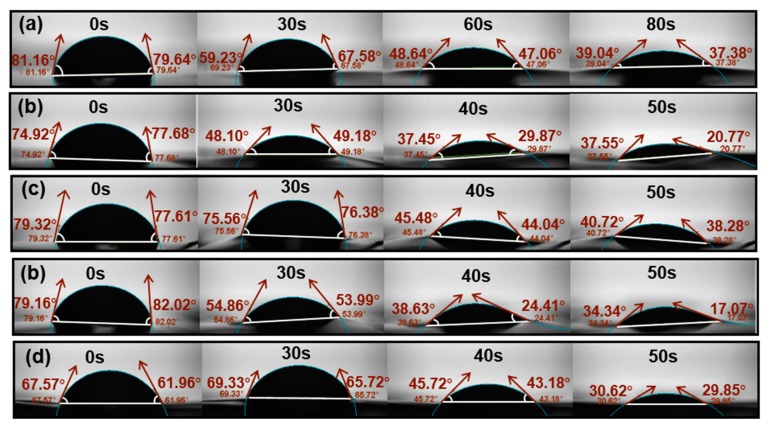
Contact angles of the blend film and plasticized blend films: (**a**) C_3_P_7_, (**b**) 20%-Gly, (**c**) 20%-TEA, (**d**) 20%-EG, and (**e**) 20%-PEG.

The water contact angles (θ) of the plasticized CP/PVA blend films were then measured to determine the effect that the four plasticizers have on the moisture sensitivity of each blend film. The photographic images in Figure 8 show the contact angles of the plasticized films were reduced to 20°–40° in a shorter time than that required for C_3_P_7_, which indicates that the addition of plasticizers increased the water sensitivities of the blend films. More specifically, the contact angle of C_3_P_7_ was reduced by just over 50% in 80 s, while the contact angles of the plasticized blend films decreased by similar amounts or slightly more in 50 s. In addition, the plasticized CP/PVA blend films exhibited varying degrees of relaxation and lifting during contact with deionized water. This phenomenon was attributed to the gradual infiltration of water droplets into the film.

**Figure 9:**
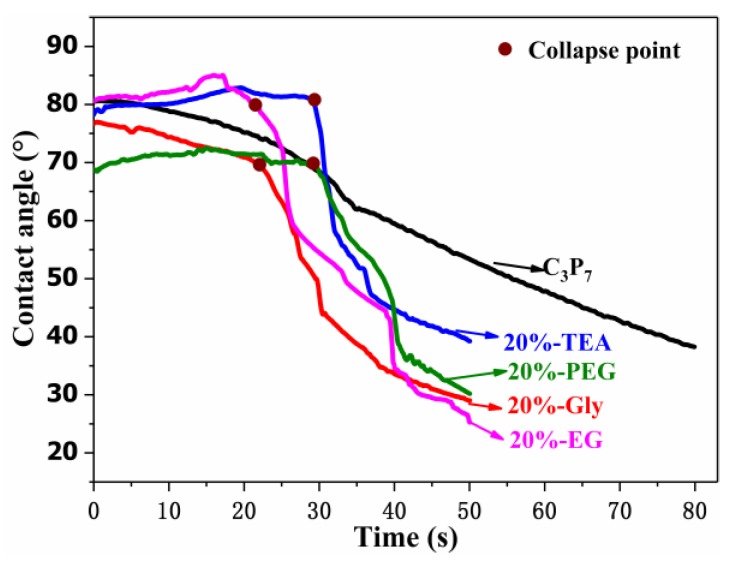
Variations in the contact angles of the blend film and plasticized blend films with time.

The variations observed in the contact angles of the blend film and plasticized blend films at different times are presented in Figure 9. For C_3_P_7_, deionized water slowly penetrated the film, gradually lowering the contact angle within 80 s. For the plasticized blend films, the water droplets collapsed when the films had been in contact with the deionized water droplets for 25–35 s. In the subsequent 5–10 s, the contact angles decreased by more than 50%. This difference in behavior between the blend film and plasticized blend films indicates that the moisture sensitivity increased in the presence of a plasticizer. A previous study has reported a similar result for Tris-plasticized FK/PVA blend films [25].

In addition, the collapse point of the 20%-TEA and 20%-PEG water droplets occurred after approximately 30 s, which was 7 s later than for 20%-Gly and 20%-EG. In the later stages of water droplet collapse (after 50 s of contact), the contact angle for 20%-TEA was still nearly 40°, whereas the contact angles for the other three plasticized films were <30°. This indicates that TEA increased the water sensitivity of the CP/PVA blend films to a lesser extent than the other plasticizers.

### 3.6. Barrier Properties of the Films Prepared

The barrier properties of a film are of particular importance when considering their potential for application in packaging materials. For example, packaging materials that exhibit a good barrier performance can effectively prevent small molecules (e.g., gas and water vapor) and microorganisms from entering the package, which ensures the stability of the internal packaging environment and prolongs the shelf life of the product. The suitability of each CP/PVA blend film for applications in packaging was, therefore, considered in terms of barrier properties, through selecting the WVP and OP of the blend films for examination.

#### 3.6.1. Water Vapor Permeability (WVP)

The process of water vapor transfer in films depends on the hydrophilic–hydrophobic balance within the matrix, and on the final film microstructure [43]. Table 2 lists the average values of water vapor permeabilities of the plasticized blend films, where it is apparent that the addition of plasticizers to the CP/PVA blend films increased the WVP, and that an increased plasticizer content further enhanced the WVP. This trend can be expected from the increase in hydrophilicity and a strengthening in the matrix continuity for mass transfer [44]. More specifically, the addition of plasticizers introduces a large number of hydrophilic groups that can improve, overall, the moisture sensitivity of the blend films. Furthermore, the continuity and number of water vapor mass transfer pathways in the blend films increased, to a certain extent, due to the formation of intermolecular interactions between the plasticizers and CP/PVA films, which expanded the free volumes of the molecular chains. Although the addition of a plasticizer can reduce the water vapor barrier, the plasticized blend film samples exhibited low WVP values that conformed to the general requirements of packaging materials [45].

#### 3.6.2. Oxygen Permeability (OP)

The OP of plasticized blend films was measured at 25 °C, 50% RH. The effects of different plasticizers and their contents on the OP of the CP/PVA blend film are also shown in Table 2. More specifically, the OP values ranged from 23.17 × 10^−5^ to 1661 × 10^−5^ cm^3^·m^−2^·d^−1^·Pa^−1^. Compared with the values obtained for the C_3_P_7_ film, the OP values significantly decreased with the addition of a plasticizer, where a greater reduction corresponded with an increased plasticizer content. In other words, the addition of a plasticizer can improve the oxygen barrier properties of the blend films, which is a trend opposite to that observed for the WVP. This is because air permeation through a film generally involves three stages; i.e., adsorption, diffusion, and desorption. Thus, the plasticizers increased the hydrophilicity of the blend film, which inhibited the adsorption of non-polar O_2_ on film surfaces [25]. Furthermore, in the presence of the plasticizer, the protein phase, characterized by a lower OP with respect to PVA, is homogeneously distributed in the PVA matrix; thus, significantly improving the barrier property in regard to oxygen. Moreover, TEA caused the largest increase in the oxygen barrier performance of the C_3_P_7_ films compared with the other plasticizers. This may be because TEA has a greater polarity compared to the other three plasticizers, which can reduce, to a greater extent, the adsorption of non-polar O_2_ on the surface of the blend membrane.

## 4. Conclusions

We, herein, reported the preparation of blend films of cottonseed protein (CP) and poly(vinyl alcohol) (PVA) using a casting method; and the mechanical properties, moisture sensitivities, and barrier properties of the plasticized blend films prepared using four different plasticizers were determined. It was found that CP/PVA mixtures, with a mass ratio of 3:7, exhibited good compatibility and no phase separation during film solidification, which we confirmed via SEM observations. In addition, Fourier transform infrared spectroscopy measurements showed that the addition of different plasticizers altered the interactions between the CP and PVA to varying degrees, and had different effects on the secondary structure of the CP. Furthermore, the addition of plasticizers to the blend enhanced the elongation at break, moisture sensitivity, and oxygen barrier properties of the films while there was a reduction in the water vapor barrier properties. Triethanolamine (TEA) was the most efficient plasticizer, which indicates that the mechanical and oxygen barrier properties of the CP/PVA blend films can be improved by the addition of TEA. This CP/PVA-TEA combination allowed a feasible method for the preparation of functional CP/PVA blend films from cheap, biodegradable, and waste CP. Moreover, this system reported herein allows the potential application of plasticized CP/PVA blend films to packaging, mulching, and other industrial processes, providing novel applications for CP. In future studies, we will exclude the possible migration and segregation of the plasticizer to eliminate the hugely detrimental effect on the properties of the material during physical aging [46] and develop new functions (such as antimicrobial and intelligent control properties of gas permeation) for CP-based blend membranes through nano- or graft-modification, which should further expand the applicability of the cottonseed protein-based composite film as a packaging material.

## Figures and Tables

**Table 1:** Secondary structure area percentages of CP in C_3_P_7_ and plasticized CP/PVA films.

Sample	Secondary Structure Area Percentage		
	β-Sheet (%)	α-Helix (%)	β-Turn (%)
C_3_P_7_	28.3	63.4	8.3
20%-Gly	27.2	61.8	11.0
20%-TEA	24.2	64.7	11.1
20%-EG	25.7	64.0	10.3
20%-PEG	26.0	63.4	10.6

**Table 2:** Water vapor permeability (WVP), oxygen permeability (OP), and thickness values for the plasticized blend films.

Sample	WVP (× 10^−12^ g·cm^−1^·s^−1^·Pa^−1^)	OP (× 10^−5^ cm^3^·m^−2^·d^−1^·Pa^−1^)	Thickness (mm)
C3P7	1.03 ± 0.05	1661 ± 8	0.06 ± 0.005
5%-Gly	1.39 ± 0.13	1072 ± 3	0.072 ± 0.007
10%-Gly	1.36 ± 0.15	381.5 ± 1.4	0.076 ± 0.004
20%-Gly	2.53 ± 0.23	75.72 ± 1.12	0.068 ± 0.005
5%-TEA	1.16 ± 0.08	1057 ± 4	0.057 ± 0.004
10%-TEA	1.5 ± 0.16	345.5 ± 1.8	0.060 ± 0.01
20%-TEA	2.04 ± 0.18	26.18 ± 0.43	0.063 ± 0.03
5%-EG	1.16 ± 0.11	1062 ± 3.2	0.073 ± 0.05
10%-EG	1.35 ± 0.12	346.3 ± 1.3	0.072 ± 0.06
20%-EG	2.63 ± 0.11	53.14 ± 0.81	0.080 ± 0.01
5%-PEG	1.22 ± 0.05	1634 ± 5	0.060 ± 0
10%-PEG	1.55 ± 0.09	98.41 ± 3.2	0.070 ± 0
20%-PEG	1.93 ± 0.17	64.01 ± 1.50	0.067 ± 0.003
